# 2229: Development of an angiogenic proteoglycan mimic to accelerate ischemic diabetic foot ulcer repair

**DOI:** 10.1017/cts.2017.31

**Published:** 2018-05-10

**Authors:** Jenny Lin, Alyssa Panitch

## Abstract

OBJECTIVES/SPECIFIC AIMS: This project aims to synthesize an angiogenic decorin mimic (VEGFp-DS-SILY) with varying densities of QK and characterize its angiogenic potential and synergism with VEGF by evaluating (1) endothelial cell (EC) migration and proliferation, (2) EC VEGF receptor activation, (3) EC tubule formation in collagen scaffolds, and (4) angiogenesis from a chick chorioallantoic membrane (CAM assay) growing into the scaffold, reflecting the ability of the collagen scaffold to integrate into existing vasculature. The next main goal is to develop and characterize an MMP-degradable nanoparticle system for controlled release of VEGF. Future work will evaluate in vivo effects of VEGFp-DS-SILY bound to a 3D collagen scaffold on ischemic wound repair in a combined excisional wound/bipedical dorsal skin flap rat model. METHODS/STUDY POPULATION: Peptide hydrazides are conjugated to the free carboxylic acid functional groups on dermatan sulfate using EDC chemistry. We added a 3 amino acid spacer (-Gly-Ser-Gly) to the C-terminus of the established QK sequence before the hydrazide functional group and refer to this modified QK as “VEGFp.” VEGFp, SILY, and *N*-terminal biotinylated versions were synthesized using standard Fmoc solid-phase peptide synthesis protocols and purified using reverse phase HPLC. Coupling efficiencies of peptides to dermatan sulfate were determined spectroscopically at 280 nm measuring the aromatic residues (Trp or Tyr) using a NanoDrop system. Dermatan sulfate with 1 or 4 VEGFp peptides coupled were termed DSV1 and DSV4, respectively. After further conjugation with SILY, we will blend this VEGFp-DS-SILY with unmodified DS-SILY to a total 10 μM to test increasing densities of VEGFp. To verify that the collagen-binding properties of VEGFp-DS-SILY are not compromised by the addition of VEGFp, we will use a streptavidin-HRP system to detect bound biotinylated VEGFp-DS-SILY on collagen-coated plates by established protocols. DSV1 and DSV4 were tested for their effects on endothelial VEGFR2 phosphorylation using an MSD ELISA-type assay and endothelial proliferation using an MTS assay. Cell migration was monitored using an ORIS assay where cells are grown to confluence around a silicone stopper that is then removed to allow cells to migrate inward. Tubulogenesis was evaluated by examining tubule formation on matrigel. Finally, in vivo angiogenesis will be evaluated using a chorioallantoic membrane assay. For extracellular VEGF release, hollow MMP-degradable thermoresponsive nanoparticles [NIPAM, 5 mol% 2-acrylamido-2-methyl-1-propanesulfonic acid (AMPS), 1% Acrylic Acid (AAc), 2 mol% MMP-degradable peptide diacrylate, and potassium persulfate initiator] will be synthesized around noncross-linked polymer cores. The cores will then be diffused out through the shell by dialysis prior to drug loading. SILY (and some biotinylated SILY for visualization) will be conjugated with EDC chemistry for targeting nanoparticles to collagen. NPs size and zeta-potential will be measured on a Malvern Zetasizer. VEGF will be loaded into NPs by co-incubating a loading solution of 1 µg/mL VEGF with 1 mg of NPs, incubating overnight at 4°C. VEGF loading and release will be measured by ELISA. Biological activity of the released VEGF from particles will be determined on ECs using assays similar to those outlined previously. RESULTS/ANTICIPATED RESULTS: Preliminary data have verified the synthesis and purification of SILY and VEGFp (QK-Gly-Ser-Gly-hydrazide), as well as an *N*-terminal biotinylated version, through mass spectrometry and reverse-phase HPLC, respectively. For proof-of-concept, we have verified binding of VEGFp to the VEGF receptor 2 using a ForteBio Blitz interferometry instrument. In addition to support based on published reports showing retained bioactivity of QK after conjugation using other spacers, our preliminary data suggests that VEGFp still binds to VEGF receptor 2, albeit with decreased affinity like QK as compared with VEGF. Circular dichroism also shows that VEGFp has retained its α-helical structure necessary for bioactivity; however it appears that it has some uncoiling when conjugated to dermatan sulfate. We hypothesize that varying densities of VEGFp conjugated to the decorin mimetic (DS-SILY) will modulate the degree of angiogenic activity and synergy with VEGF. We determined that we can achieve ~70% VEGFp conjugation completion to dermatan sulfate after 3.5 hours. We have quantified VEGFR2 phosphorylation after 5 minute treatments by using phospho-specific antibodies and an ELISA-type protocol in a mesoscale discovery system. Preliminary data with human umbilical vein endothelial cells shows that VEGFp exhibits synergism with VEGF at levels similar to QK. DSV1 and DSV4 data suggests synergy with VEGF, although free-peptides and engineered compounds alone did not show effects similar to VEGF in the conditions tested. Prelminary data with 30 minute treatments suggests that the peptides and compounds may require longer exposures to induce activation, as they may have slower binding rates. In contrast, prolonged stimulation with VEGF causes a sharp increase in receptor activation, peaking around 10 minutes and decreasing significantly by 30 minutes. Peptides QK and VEGFp both slightly increased proliferation of dermal microvascular endothelial cells (HMVECs) after 60 hours incubation. However, incubation with dermatan sulfate and DSV caused significant cell death after 24 hours in reduced growth factor media, likely due to sequestering of growth factors. It is possible that VEGFp-DS-SILY may better stimulate proliferation since it would be presented as a surface bound proteoglycan mimic, rather than as a soluble factor. HMVECs migrated farther for all treatment groups (10 µM QK, 10 µM VEGFp, 1 µM DSV4, and 10 µM DSV4) than the 10 ng/mL VEGF positive control, although more cells migrated in response to VEGF. This may be accounted at least in part by the more pronounced proliferation induced by VEGF. Migration will also be tested in 3D culture within a collagen gel. We are currently testing a 2D matrigel system for tubulogenesis. We have found that 10 µM DSV4 forms qualitatively more well-defined tubules than the untreated control on reduced growth factor matrigel. However, we were not able to quantify the improved tubule formation and are still troubleshooting the tubule analysis. After seeding ECs and culturing for 4, 8, and 12 hours, cells will be fluorescently stained with anti-CD31 and imaged for 3D tubule formation. CAM assay angiogenesis growing into a collagen scaffold. In brief, fertilized chicken embryos are incubated for 2 days before exposing the CAM. VEGFp-DS-SILY bound to a collagen gel will be placed onto the CAM. Some treatment groups will receive additional VEGF to investigate synergistic effects. Light microscope images of angiogenesis into the collagen gel coated with VEGFp-DS-SILY, taken every day from days 10 to 13, will reflect the ability of the collagen scaffold to integrate into existing vasculature and 3D angiogenic potential of VEGFp-DS-SILY with or without VEGF. We expect that VEGFp-DS-SILY treatment will increase the number of vessels formed on the CAM. Preliminary data using a Fluoraldehyde assay indicates that loading of ~300 ng VEGF per mg of nanoparticles can be achieved. We expect that using an MMP-degradable peptide diacrylate crosslinker will allow nanoparticles to degrade in protease-rich environments like the chronic wound bed and release VEGF. Adjustments to the formulation, such as crosslinker density, may need to be modified to control the rate of VEGF release. DISCUSSION/SIGNIFICANCE OF IMPACT: We expect that our angiogenic decorin mimetic will lead to a novel treatment to accelerate healing of ischemic diabetic foot ulcers, thereby reducing the need for limb amputation and mortality rate of diabetic patients. We anticipate that the diabetes research and regenerative medicine communities will (1) gain a platform for targeted delivery of growth factors, (2) understand the dependence of vascularization within 3D collagen constructs on VEGFp densities and VEGF receptor activation in controlling the degree of angiogenesis, and (3) gain the benefits of controlled angiogenesis in ischemic diabetic wound healing.

